# Disposal of children’s stools and its association with childhood diarrhea in India

**DOI:** 10.1186/s12889-016-3948-2

**Published:** 2017-01-05

**Authors:** Rahul Bawankule, Abhishek Singh, Kaushalendra Kumar, Sarang Pedgaonkar

**Affiliations:** 1 International Institute for Population Sciences, Mumbai, India; 2 Department of Public Health & Mortality Studies, International Institute for Population Sciences, Mumbai, India; 3 Department of Population Policies and Programmes, International Institute for Population Sciences, Mumbai, India

**Keywords:** Children’s stool disposal, Childhood diarrhea, India

## Abstract

**Background:**

Children’s stool disposal is often overlooked in sanitation programs of any country. Unsafe disposal of children’s stool makes children susceptible to many diseases that transmit through faecal-oral route. Therefore, the study aims to examine the magnitude of unsafe disposal of children’s stools in India, the factors associated with it and finally its association with childhood diarrhea.

**Methods:**

Data from the third round of the National Family Health Survey (NFHS-3) conducted in 2005–06 is used to carry out the analysis. The binary logistic regression model is used to examine the factors associated with unsafe disposal of children’s stool. Binary logistic regression is also used to examine the association between unsafe disposal of children’s stool and childhood diarrhea.

**Result:**

Overall, stools of 79% of children in India were disposed of unsafely. The urban-rural gap in the unsafe disposal of children’s stool was wide. Mother’s illiteracy and lack of exposure to media, the age of the child, religion and caste/tribe of the household head, wealth index, access to toilet facility and urban-rural residence were statistically associated with unsafe disposal of stool. The odds of diarrhea in children whose stools were disposed of unsafely was estimated to be 11% higher (95% CI: 1.01–1.21) than that of children whose stools were disposed of safely. An increase in the unsafe disposal of children’s stool in the community also increased the risk of diarrhea in children.

**Conclusion:**

We found significant statistical association between children’s stool disposal and diarrhea. Therefore, gains in reduction of childhood diarrhea can be achieved in India through the complete elimination of unsafe disposal of children’s stools. The sanitation programmes currently being run in India must also focus on safe disposal of children’s stool.

## Background

The disposal of children’s stools has received little attention so far in the sanitation programmes of any country and, in fact, stools of young babies and children are regarded harmless and not dirty in many societies [1]. Interestingly, the sanitation programmes usually focus on household sanitation and overlook disposal practices of children’s stool [2]. According to the World Health Organization (WHO), a child’s stool is considered to be disposed of safely when the child uses the toilet/latrine; the faeces is put/rinsed in the toilet/latrine or buried. On the contrary, the disposal of stool is considered unsafe if the faeces is put/rinsed in a drain/ditch, thrown in the garbage, left or buried in the open [3]. In the Indian subcontinent, unsafe disposal of the stools of under-three children is more prevalent in Bangladesh (78%), Nepal (69%) and Afghanistan (52%) [2, 4, 5], with India, not far behind. A recent study from rural Odisha reported that the stools of 90% of preambulatory and 70% of ambulatory children were disposed of unsafely [6]. Despite high levels of unsafe disposal, there is hardly any study that has examined the underlying socioeconomic and demographic determinants of unsafe disposal of children’s stool in India.

Children’s stools are a more dangerous source of faecal contamination in the household environment because children are more prone to be exposed to fecal pathogens than adults due to their behaviors [3, 7]. While crawling or playing, children may put contaminated fingers, pica or fomites into their mouth [8]. In India, children usually defecate in the compound or in and around the house on the ground and stools are usually left open [9]. Utensils, hands, and water can be easily contaminated by faecal pathogens and cause faecal-oral diseases [7]. Children are more susceptible to faecal-oral diseases, and thus, unsafe disposal of children’s stool carries a higher health risk [10].

Diarrhea is the most common faecal-oral disease in children and is defined by the WHO as the passage of unusually loose or watery stool, usually at least three times in a twenty-four hour period [11]. Diarrhea is mostly infectious in nature [12]. Childhood diarrhea is the second leading cause of under-five mortality in the world. Estimates suggest that childhood diarrhea kills 760,000 children annually in the world [13]. Globally, diarrheal diseases contribute to 4% of the total disease burden, 3% of the overall mortality and 11% of the under-five mortality. In India, where the highest number of infant and child deaths occur, childhood diarrheal mortality is also the highest in the world [14–16]. Million Death Study Collaborators reported that diarrhea accounted for 26% of all under-five deaths (about 1.3 million) in India during 2001–03 [17].

Given the serious consequences of unsafe disposal of children’s stools, a few epidemiological studies have reported the linkage of childhood diarrhea with the disposal of children’s stools. Children whose stools are disposed of unsafely have a higher risk of diarrhea than those whose stools are disposed of safely, and the odds of diarrhea range from a minimum of 1.3 to a maximum of 2.2 [18–21]. In Sri Lanka, children from households where excreta was disposed of in a latrine were less likely to have diarrhea than children whose families disposed of excreta improperly [22]. A study from Iraq reported that children whose stools were put in a latrine/toilet were less likely to suffer from diarrhea than those whose stools were left in the open [23]. Indiscriminate disposal of stools was one of the risky behaviors of mothers causing diarrhea in children [24]. Studies from Ghana and India found that neighborhood sanitation and environment had a significant effect on child health and mortality [25, 26]. Similarly, past studies have shown that children aged 6–23 months, male children and those born with low weight are at higher risk of diarrhea [19, 20, 23, 27–29]. Use of unimproved toilet facilities and use of an unimproved source of drinking water increase children’s susceptibility to diarrhea [29, 30]. Besides, mother’s illiteracy and lower age (<25 years), the poor economic status of household and rural residence are also found to be associated with childhood diarrhea [19, 20, 28–31].

India is a large and diverse country where open defecation is very common and has highest under-five child deaths associated with diarrhea globally [16, 32]. However, to date, there is no national or state representative study that has investigated the effect of disposal of children’s stools on childhood diarrhea. Perhaps, disposal of children’s stools itself is an under-researched subject in India. Given the lack of systematic studies on the subject, our study aims to examine the magnitude of unsafe disposal of children’s stools in India, the factors associated with it and the prevalence of diarrhea in children below five years of age. Finally, our study investigates the association between disposal of children’s stool and childhood diarrhea after adjusting for selected socioeconomic and demographic factors. Also, we examine the association between diarrhea in children and unsafe disposal of children’s stool in the neighborhood.

## Methods

### Study design and setting

The study is cross-sectional in nature and based on data from population-based National Family Health Survey 2005–06 (NFHS-3), India. NFHS-3 is a nationally representative population-based cross-sectional survey conducted across 29 states of India in 2005–06. The main objective of NFHS-3 was to provide national and state estimates of fertility, family planning, infant and child mortality, reproductive and child health, nutrition of women and children, and the quality of health and family welfare services. In India, NFHS-3 is the first survey that collected information on children’s stool disposal practices.

NFHS-3 adopted a multistage sampling design – a two stage design in rural areas and a three stage design in urban areas for selecting households and eligible men and women for an interview. Altogether, 109,041 households, 124,385 women aged 15–49 years and 74,369 men aged 15–54 years were interviewed in the survey. The response rates were 98, 94, and 87%, respectively [33, 34].

### Inclusion/exclusion criteria

The information on diarrhea was collected for all children born during the five years preceding NFHS-3. The question related to disposal of stool was asked with respect to the youngest child born in the five years preceding NFHS-3. Hence, we included only the youngest child from each household in the analysis.

### Ethics statement

The analysis presented in the paper is based on National Family Health Survey 2005–06 (NFHS-3) which is a publically available dataset with no identifiable information on the survey participants. All the ethical concerns, including informed consent, are strictly followed in the NFHS-3. Given these, no ethical approval or informed consent was required for the current study.

### Variables

To get details about the disposal of children’s stools, mothers of under-five children were asked, “The last time (NAME OF YOUNGEST CHILD) passed stools, what was done to dispose of the stools”? The response categories to this questions included, ‘child used the toilet or latrine,’ ‘put/rinsed into toilet or latrine,’ ‘put/rinsed into drain/ditch,’ ‘thrown into the garbage,’ ‘buried,’ ‘left in the open,’ and ‘other.’ Based on the WHO/UNICEF definition, we generated a new variable, ‘children’s stool disposal’ which has only two categories. The categories ‘child used toilet or latrine’, ‘put/rinsed into toilet or latrine’ and ‘buried’ were combined and coded as ‘safe disposal of stool.’ The others were coded as ‘unsafe disposal of stool’ [3]. Finally, the disposal of children’s stools was coded as ‘0’ if the stool was disposed of safely, and ‘1’ otherwise.

To examine the factors associated with the disposal of children’s stools, age and sex of children, mother’s age, literacy (literate, illiterate) and working status of mother (currently working for cash/kind, not working), mother’s exposure to media, religion and caste/tribe of household head, household’s wealth, presence of improved toilet facility in the household and place of residence (urban, rural) were included in the analysis. The wealth index computed in NFHS-3 included 33 household assets including toilet facility and source of drinking water. Since we wanted to examine the independent effect of toilet facility and source of drinking water on the practice of stool disposal and prevalence of diarrhea, we generated a new wealth index which did not include toilet facility and source of drinking water. We followed the procedure of NFHS-3 to generate the new wealth index. The new wealth index was further categorized into lowest one-third (lowest), middle one-third (middle) and highest one-third (highest). The variable on media exposure includes exposure to newspaper, television, and radio. The mothers who were exposed to all the three media were coded as ‘fully exposed.’ Those who were exposed to one or two sources of media were coded as ‘partially exposed.’ The rest were coded as ‘not exposed.’ Further, the toilet facility and source of drinking water were categorized into ‘improved’ and ‘unimproved’ following the WHO/UNICEF definition [3].

For assessing the occurrence of diarrhea in children below five years of age, mothers were asked ‘Has (NAME) had diarrhea in the last two weeks?’ The diarrhea was coded as ‘0’ if the mother reported ‘no’ and ‘1’ otherwise. The key independent variable in the analysis of the occurrence of diarrhea is the disposal of children’s stool. We also estimated a variable on unsafe disposal of children’s stool in the neighborhood. This variable was estimated by taking the mean of unsafe disposal of children’s stool over all children in a Primary Sampling Unit (PSU) other than the index child. Age and sex of children, maternal age, literacy and working status of the mother, mother’s exposure to media, religion and caste/tribe of household head, household’s wealth, the source of drinking water, toilet facility and birth size (larger, average, smaller) were also included in the analysis. In NFHS-3 mothers were asked to report the size of the baby at birth. The response categories were very large, larger than average, average, smaller than average and very small. The babies reported as ‘very large’ or ‘larger than average’ were coded as ‘larger.’ The babies reported as ‘average’ were coded as ‘average’ and those reported as ‘smaller than average’ and ‘very small’ were coded as ‘smaller.’ We also estimated a variable namely percentage of households in the neighborhood using unimproved toilet or practicing open defecation. This variable was estimated by taking the mean of the households using unimproved toilet or practicing open defecation over all households in a PSU other than the index household.

### Statistical analysis

We used bivariate and multivariate analysis to fulfill the objectives of the paper. F-test was used in the bivariate analysis to examine the association between the dependent and the independent variables. We used a multivariable binary logistic regression model to examine the factors associated with unsafe disposal of children’s stool and to examine the association between disposal of children’s stools and the occurrence of diarrhea in children.

The analysis was carried out in STATA 13.0. Appropriate sampling weights were used in the estimations. We clustered standard errors at the PSU level to take into account the survey design of NFHS-3. All the independent variables were tested for possible multicollinearity before putting those into the regression models.

## Results

Table 1 shows the percentage of unsafe disposal of children’s stool by selected socioeconomic and demographic characteristics. Overall, the stool of nearly 79% of children was disposed of unsafely. The urban-rural gap in the unsafe disposal of stool was wide. The stools of nearly 88% of the children in rural areas were disposed of unsafely compared with 54% in urban areas. Strikingly, the disposal of stools of more than 50% of the children was unsafe even in urban India.

**Table 1:** Unsafe disposal of children’s stool by socioeconomic and demographic characteristics, India, 2005-06

Socioeconomic and demographic characteristics	Unsafe (%)	*P*-value	Number
Toilet facility			
Improved	53.8	0.000	11,909
Unimproved	86.8		23,364
Age of mother (in years)			
20–34	76.6	0.000	27,663
15–19 and 35–49	86.0		7610
Mother’s literacy			
Non-Literate	91.2		13,368
Literate	67.7	0.000	21,905
Mother’s working status			
Not working	76.0	0.000	24,894
Working	85.2		10,379
Mother’s exposure to media			
No	92.5	0.000	8023
Partial	86.4		9265
Full	63.7		17,985
Age of child (in months)			
0–5	88.0	0.000	4619
6–11	87.0		4859
12–17	85.2		4733
18–59	73.3		21,062
Sex of child			
Male	78.0	0.003	18,921
Female	80.0		16,352
Religion			
Hindu	80.7	0.000	24,683
Muslim	74.5		5,610
Other religion	61.0		4,980
Caste/Tribe			
Scheduled Caste (SC)	86.8	0.000	6056
Scheduled Tribe (ST)	89.7		5463
Non SC/ST	75.1		23,754
Wealth index			
Lowest	94.0	0.000	13,918
Middle	78.0		11,553
Highest	44.7		9802
Place of residence			
Urban	52.6	0.000	14,012
Rural	88.5		21,261
Total	78.7		35,273

All values in the table represent absolute numbers and percentages unless otherwise stated

Percentages are weighted and Ns are unweighted

The results of the multivariable binary logistic regression assessing the factors associated with unsafe disposal of the stool are presented in Table 2. Lack of access to improved toilet facility was statistically associated with unsafe disposal of stool. The odds of disposing of stools unsafely among households having a lack of access to improved toilets were twice (AOR: 2.20; 95% CI: 2.08–2.33) that of households having access to improved toilets. The odds of disposing of the stools unsafely were 43% lower (AOR: 0.57; 95% CI: 0.53–0.61) in literate mothers than illiterate mothers. Unsafe disposal of stool also depended on the mother’s exposure to media. The odds of unsafe stool disposal was 37% lower (AOR: 0.63; 95% CI: 0.57–0.70) in mothers who were fully exposed to media than in mothers who were not exposed to media. Similarly, the odds of disposing of the stools unsafely were 18% lower (AOR: 0.82; 95% CI: 0.75–0.91) in mothers who were partially exposed to media than those who were not exposed to media. Age of the mother and mother’s work were not associated with unsafe disposal of stool in the regression model.

**Table 2:** Results of multivariable binary logistic regression showing the risk of unsafe stool disposal, India, 2005–06

Socioeconomic and demographic characteristics	Adjusted Odds Ratio (95% CI)
Toilet facility	
Improved®	
Unimproved	2.20*(2.08,2.33)
Age of mother (in year)	
20–34®	
15–19 and 35–49	1.06(0.99,113)
Mother’s literacy	
Non-literate®	
Literate	0.57*(0.53,0.61)
Mother’s working status	
Not working®	
Working	0.99(0.93,1.06)
Mother’s exposure to media	
No®	
Partial	0.82*(0.75,0.91)
Full	0.63*(0.57,0.70)
Age of child (in months)	
0–5®	
6–11	3.51*(3.20,3.85)
12–17	3.19*(2.92,3.48)
18–59	2.49*(2.29,2.72)
Sex of child	
Male®	
Female	1.00(0.95,1.05)
Religion	
Hindu®	
Muslim	0.69*(0.64,0.75)
Other religion	0.44*(0.40,0.48)
Caste/Tribe	
Scheduled Caste (SC)®	
Scheduled Tribe (ST)	1.45*(1.34,1.57)
Non SC/ST	0.97(0.88,1.07)
Wealth index	
Lowest®	
Middle	0.50*(0.46,0.54)
Highest	0.23*(0.21,0.26)
Place of residence	
Urban®	
Rural	2.42*(2.28,2.57)

*CI* confidence interval; *p-value < 0.05

Unsafe disposal of stool was also statistically associated with the religion of the head of the household. The odds of unsafe disposal were 31% lower (AOR: 0.69; 95% CI: 0.64–0.75) among Muslim households than Hindu households. Likewise, the odds of disposing of stools unsafely among households from ‘Other religion’ were 56% lower (AOR: 0.44; 95% CI: 0.40–0.48) than Hindu households. Another variable that was statistically associated with unsafe disposal of stool was the household’s wealth index. The odds of unsafe disposal among middle one-third households was half (AOR: 0.50; 95% CI: 0.46–0.54) that of the lowest one-third households. Likewise, the odds of disposing of stools unsafely was 77% lower (AOR: 0.23; 95% CI: 0.21–0.26) in highest one-third households than in lowest one-third households. The odds of unsafe disposal among households residing in rural areas were twice (AOR: 2.42; 95% CI: 2.28–2.57) compared to households residing in urban areas.

Table 3 presents the prevalence of diarrhea in children below five years of age by selected socioeconomic and demographic characteristics. Overall, 11% of children below five years of age suffered from diarrhea. The prevalence of diarrhea did not vary by urban-rural residence. Age of mother, mother’s exposure to media, the age of child, sex of child, size of the child at birth, religion, and wealth index were statistically associated with the prevalence of diarrhea in the bivariate analysis.

**Table 3:** Prevalence of diarrhea in children below 5 years of age by socioeconomic and demographic characteristics, India, 2005–06

Socioeconomic and demographic characteristics		Prevalence (%)	*P*-value	Number
Children’s stool disposal	Safe	9.0	0.000	11,202
	Unsafe	11.2		24,067
Toilet facility	Improved	9.6	0.008	11,907
	Unimproved	11.0		23,362
Source of drinking water	Improved	10.6	0.321	27,287
	Unimproved	11.1		7981
Age of child (in months)	0–5	10.5	0.000	21,058
	6–11	18.1		4619
	12–17	15.7		4859
	18–59	7.9		4733
Sex of child	Male	11.1	0.045	18,919
	Female	10.3		16,350
Size of child at birth	Larger	10.5	0.000	8104
	Average	9.8		19,576
	Smaller	13.1		7588
Age of mother (in years)	20–34	10.4	0.005	27,659
	15–19 and 35–49	11.8		7610
Mother’s literacy	Non-literate	10.9	0.357	21,902
	Literate	10.5		13,367
Mother’s working status	Not working	11.1	0.005	24,890
	Working	9.8		10,379
Mother’s exposure to media	No	11.0	0.190	8023
	Partial	11.1		9265
	Full	10.2		17,981
Religion	Hindu	10.4	0.043	24,681
	Muslim	12.0		5608
	Other religion	11.1		4980
Caste/Tribe	SC	10.7	0.946	6056
	ST	10.9		5463
	Non SC/ST	10.7		23,750
Wealth index	Lowest	11.0	0.058	13,918
	Middle	11.0		11,551
	Highest	9.7		9800
Place of residence	Urban	10.3	0.258	14,009
	Rural	10.9		21,260
Total		10.7		35,269

All values in the table represent absolute numbers and percentages unless otherwise stated. Percentages are weighted and Ns are unweighted

We estimate three separate multivariable binary logistic regression models to examine the association between unsafe disposal of children’s stool and diarrhea in children (Table 4). The first model included disposal of children’s stool, the source of drinking water, children and mother related and household related characteristics. The results indicate a statistically significant association between unsafe disposal of children’s stool and diarrhea in children. The odds of suffering from diarrhea was 11% higher (AOR: 1.11; 95% CI: 1.01–1.21) in children whose stools were disposed of unsafely compared to children whose stools were disposed of safely. The results adjusted for stool disposal of neighborhood children (Model 2) indicate a statistically significant association between unsafe disposal of children’s stool in the neighborhood and diarrhea in children. A unit increase in the proportion of unsafe disposal of children’s stool in the neighborhood was associated with a 1% higher risk of diarrhea in children (AOR: 1.01; 95% CI: 1.00–1.01). The effect of unsafe disposal of children’s stool was lost the moment we included the variable on unsafe disposal of children’s stool in the neighborhood in the regression model. We also included the use of toilet facility and proportion of households using the unimproved toilet or practicing open defecation in the neighborhood in Model 3. Unsafe disposal of children’s stool in the neighborhood was statistically associated with higher risk of diarrhea in children. This finding clearly indicates that the unsafe disposal of children’s stool in the neighborhood has a much larger association with diarrhea than the unsafe disposal of index child’s stool. The other variables that were associated with diarrhea in children in multivariable binary logistic regression analysis are the age of the child, sex of the child, the size of the child at birth, mother’s literacy and proportion of households using the unimproved toilet in the community.

**Table 4:** Results of multivariable binary logistic regression showing the risk of diarrhea in children below 5 years of age, India, 2005–06

Socioeconomic and demographic characteristics		Adjusted Odds Ratio (95% CI)		
		Model 1	Model 2	Model 3
Children’s stool disposal	Safe®			
	Unsafe	1.11*(1.01,1.21)	1.04(0.94,1.14)	1.02(0.93,1.12)
Unsafe disposal of children’s stool in the neighborhood (%)		–	1.01*(1.00,1.01)	1.01*(1.00,1.01)
Percentage of households in the neighborhood using unimproved toilet or practicing open defecation		–	–	1.00*(0.99,1.01)
Toilet facility	Improved®	–	–	
	Unimproved	–	–	0.99(0.89,1.09)
Source of drinking water	Improved®			
	Unimproved	0.99(0.91,1.07)	0.98(0.90,1.06)	0.98(0.90,1.06)
Age of child (in months)	0–5®			
	6–11	1.84*(1.64,2.08)	1.85*(1.64,2.08)	1.84*(1.64,2.08)
	12–17	1.52*(1.34,1.72)	1.52*(1.34,1.72)	1.52*(1.34,1.72)
	18–59	0.77*(0.69,0.86)	0.77*(0.69,0.85)	0.77*(0.69,0.85)
Sex of child	Male®			
	Female	0.90*(0.84,0.96)	0.90*(0.84,0.96)	0.90*(0.84,0.96)
Size of child at birth	Large			
	Average	0.88*(0.81,0.96)	0.87*(0.80,0.95)	0.88*(0.80,0.95)
	Small	1.24*(1.13,1.37)	1.24*(1.13,1.37)	1.24*(1.13,1.37)
Age of mother (in year)	20–34®			
	15–19 and 35–49	1.02(0.94,1.10)	1.01(0.93,1.10)	1.01(0.93,1.10)
Mother’s literacy	Non-literate®			
	Literate	1.10*(1.01,1.20)	1.13*(1.04,1.23)	1.14*(1.05,1.24)
Mother’s working status	Not working®			
	Working	1.01(0.93,1.09)	1.01(0.93,1.09)	1.01(0.93,1.09)
Mother’s exposure to media	No®			
	Partial	1.06(0.96,1.18)	1.07(0.97,1.18)	1.07(0.97,1.19)
	Full	1.01(0.90,1.12)	1.03(0.92,1.15)	1.03(0.92,1.15)
Religion	Hindu®			
	Muslim	1.14*(1.03,1.25)	1.12*(1.01,1.23)	1.13*(1.03,1.24)
	Other religion	1.03(0.92,1.16)	1.05(0.93,1.17)	1.08(0.96,1.21)
Caste/Tribe	SC®			
	ST	0.93(0.82,1.07)	0.93(0.82,1.06)	0.94(0.82,1.07)
	Non SC/ST	0.96(0.88,1.06)	0.96(0.87,1.06)	0.96(0.88,1.06)
Wealth index	Lowest®			
	Middle	0.97(0.88,1.06)	1.00(0.91,1.10)	1.00(0.91,1.10)
	Highest	0.89(0.79,1.00)	0.92(0.82,1.04)	0.95(0.84,1.07)
Place of residence	Urban®			
	Rural	0.97(0.90,1.06)	0.92(0.84,1.00)	0.90*(0.83,0.99)

*CI* confidence interval; *p-value < 0.05

## Discussion

Our study is perhaps the first in India that has examined the socioeconomic and demographic determinants of unsafe disposal of children’s stools. It is also the first study in India that uses a large-scale population-based representative dataset to investigate the association between unsafe disposal of stool and diarrhea in children. Overall, the stool of 79% of children below five years of age was disposed of unsafely. Being non-literate, having lower exposure to media, belonging to Hindu religion, belonging to scheduled castes/tribes, having lower wealth quintile, having access to unimproved toilet facility and being a rural resident were the factors associated with unsafe disposal of children’s stool. A study in Burkina Faso also suggested a significant association of wealth and education with hygienic behavior [35]. Curtis et al. (2001) suggested that health promotion programmes, through health education and mass media, resulted in 4% increase in safe disposal of stool in Burkina Faso [36]. A study conducted in Ethiopia also reported similar findings [37]. Our findings are also consistent with the findings of a recent study conducted in rural Odisha [6].

Our study confirms the association between disposal of children’s stools and diarrhea in children below five years of age. Children whose stools were disposed of unsafely were more likely to suffer from diarrhea than children whose stools were disposed of safely. A few studies conducted in different settings support our findings [18–21]. We further found that children were more likely to have diarrhea if stool of children residing in the neighborhood was disposed of unsafely. This finding is also consistent with the studies from Ghana and India, where household externalities played an important role in child health and mortality [25, 26]. The other variables that were associated with diarrhea in children in multivariable binary logistic regression analysis are the age of the child, sex of the child, the size of the child at birth and mother’s literacy. Studies conducted in Sub-Saharan Africa, Iraq, and Turkey support our findings [20, 23, 28, 29, 38].

Our findings have important policy implications. A key finding of our study is that increased access to improved toilets is likely to reduce the unsafe disposal of stool in India. In this context, our study lends support to the ‘Swachh Bharat Mission,’ which emphasizes making available basic sanitation facilities to all Indians by 2019 [39]. The mission aims to build 66575 household latrines per day to cover all households in five years. However, our findings show that even in households that have access to improved toilets, the disposal of stools of 54% percent of the children is unsafe. This indicates that just having access to improved toilets does not ensure utilization of the toilet facility for disposing of the children’s stool. Moreover, in nearly 33% of rich households, stools of 45% of the children were disposed of unsafely. Also, the effective disposal of child feces is an essential indicator for open defecation free certification under the Swachh Bharat Mission. A document prepared by the World Sanitation Programme reports that despite a few interventions, there is still not a strong evidence base of effective strategies for reducing the unsafe disposal of child feces [40]. Our findings call for strategies that go beyond building toilets. Strategies such as motivating and educating mothers on safe disposal of children’s stools along with building toilets can go a long way in curbing the unsafe disposal of children’s stools in India. Mothers who do not have access to improved toilet facility must be informed on the safe burial of children’s stools.

Our findings show that increase in maternal education and media exposure are likely to reduce the unsafe disposal of children’s stools. These findings indicate that health promotional activities must make active use of Information Education and Communication (IEC) and mass media. Village Health, Sanitation, and Nutrition Committees (VHSNC) set up under the National Rural Health Mission (NRHM) need to be strengthened, trained and made functional for creating awareness among communities about the safe disposal of stools. Community health workers like Accredited Social Health Activists (ASHA) and Anganwadi Workers (AWW) may be engaged to educate women on safe disposal of stools.

### Strengths and limitations of study

The strengths and limitations of the study must be noted. One of the major strengths is the use of a nationally representative population-based dataset for examining the method of disposal of children’s stools and its effect on diarrhea in the Indian context. In fact, NFHS-3 is the first and only large-scale survey in India, which collected information on practices regarding disposal of stools.

A key limitation of the study is that the data used in the analysis dates back to 2005–06. The Government of India has built latrines under various programs over this period [39]. However, we would like to mention that NFHS-3 is the most recent large-scale household survey that has collected information on children’s stool disposal and prevalence of diarrhea in India. Also, the NFHS-3 reported that 29% of the households in India had access to improved toilet in 2005–06 [33]. The latest Indian Census (conducted in India in 2011) shows that about 30% of the households in India have access to improved toilet [41]. These data, thus, suggest that there is not much difference in the access to improved toilet in NFHS-3 and recent estimates obtained from the Indian Census. Moreover, the underestimation of access to improved toilet, if at all present in NFHS-3, should not affect the relationship between children’s stool disposal and occurrence of diarrhea in India as studies have shown that access to improved toilet is not a guarantee that the children’s stool will be disposed of safely [42, 43]. Another limitation of the study is that we could not adjust the results for the effect of washing hands on diarrhea because the information on washing hands was not collected in NFHS-3. We also could not adjust the odds ratio for birth weight in the diarrhea analysis. However, we did adjust the odds ratio for the size of the baby at birth. In settings where birth weight is not available for all children, the size of children at birth is a good proxy for birth weight. According to the NFHS-3, about 66% of the children below five years of age were not weighed at birth [33]. Another limitation of our study relates to the way in which information on disposal of stools was collected in NFHS-3. The NFHS-3 relied on reported practice rather than direct observation. While Gil et al. (2004) found greater precision in studies employing structured observations [44], Clasen et al. (2012) found that direct observations are subject to reactivity (Hawthorne effect) in the study population [43]. Reporting bias and recall lapse in our study is likely to be least because the NFHS-3 enquired about the ‘last time’ rather than the usual practice of disposal of stools. Again, the measurement of the prevalence of diarrhea in NFHS-3 is based on a 14-day recall period. Manesh et al. (2008) reported significant recall and reporting bias in childhood morbidities in Demographic and Health Surveys (DHS) [45]. Similarly, Schmidt et al. (2011) argued that DHSs should collect point prevalence rather than the period prevalence of diarrhea [46]. However, all the DHSs conducted in India and abroad and the UNICEF sponsored surveys in India use a 14-day recall period to measure the prevalence of diarrhea in children.

## Conclusion

Despite these limitations, this study brings to the forefront the magnitude of unsafe disposal of children’s stools in India. This study also provides empirical evidence on the association between unsafe disposal of children’s stool in a community and diarrhea in children. Our study raises a number of questions about the effectiveness of the sanitation programmes being run in India. Given the serious consequences of unsafe disposal of children’s stool, there is a need to redesign the sanitation programmes being run in India. Children remain vulnerable to diarrhea till the stools of all children in the neighborhood are not disposed of safely. Thus, inclusion of strategies to reduce unsafe disposal of children’s stools in the sanitation programmes can go a long way in reducing the risk of diarrhea in children below five years of age.

## Abbreviations

AOR: Adjusted odds ratio
ASHA: Accredited social health activists
AWW: Anganwadi workers
CI: Confidence interval
DHS: Demographic and health surveys
IEC: Information education communication
NFHS-3: National family health survey (3^rd^ round)
NRHM: National rural health misson
PSU: Primary sampling unit
UNICEF: United Nations children’s fund
VHSNC: Village health, sanitation, and nutrition committee
WHO: World health organization
